# Whole-Genome Sequencing Analysis of Human Metabolome in Multi-Ethnic Populations

**DOI:** 10.1038/s41467-023-38800-2

**Published:** 2023-05-30

**Authors:** Elena V. Feofanova, Michael R. Brown, Taryn Alkis, Astrid M. Manuel, Xihao Li, Usman A. Tahir, Zilin Li, Kevin M. Mendez, Rachel S. Kelly, Qibin Qi, Han Chen, Martin G. Larson, Rozenn N. Lemaitre, Alanna C. Morrison, Charles Grieser, Kari E. Wong, Robert E. Gerszten, Zhongming Zhao, Jessica Lasky-Su, NHLBI Trans-Omics for Precision Medicine (TOPMed), Honghuang Lin, Jeffrey Haessler, Jennifer A. Brody, Kari E. North, Kent D. Taylor, Clary B. Clish, James G. Wilson, Xihong Lin, Robert C. Kaplan, Charles Kooperberg, Bruce M. Psaty, Stephen S. Rich, Jerome I. Rotter, Ramachandran S. Vasan, Eric Boerwinkle, Bing Yu

**Affiliations:** 1 Human Genetics Center, School of Public Health, The University of Texas Health Science Center, Houston, TX USA; 2 Center for Precision Health, School of Biomedical Informatics, The University of Texas Health Science Center at Houston, Houston, TX USA; 3 Department of Biostatistics, Harvard T.H. Chan School of Public Health, Boston, MA USA; 4 Division of Cardiovascular Medicine, Beth Israel Deaconess Medical Center, Harvard Medical School, Boston, MA USA; 5 Department of Biostatistics and Health Data Science, Indiana University School of Medicine, Indianapolis, IN USA; 6 Channing Division of Network Medicine, Department of Medicine, Brigham and Women’s Hospital and Harvard Medical School, Boston, MA USA; 7 Retina Service, Massachusetts Eye and Ear, Harvard Medical School, 243 Charles Street, Boston, MA USA; 8 Department of Epidemiology and Population Health, Albert Einstein College of Medicine, Bronx, NY USA; 9 Department of Biostatistics, Boston University School of Public Health, Boston, MA USA; 10 Cardiovascular Health Research Unit, Departments of Medicine, Epidemiology, and Health Systems and Population Health, University of Washington, Seattle, WA USA; 11 Metabolon Inc., Morrisville, NC USA; 12 Division of Public Health Sciences, Fred Hutchinson Cancer Research Center, Seattle, WA USA; 13 Broad Institute of Harvard and MIT, Cambridge, MA USA; 14 Department of Medicine, University of Massachusetts Medical School, Worcester, MA USA; 15 Department of Epidemiology, University of North Carolina Gilling School of Global Public Health, Chapel Hill, NC USA; 16 Carolina Center of Genome Sciences, University of North Carolina, Chapel Hill, NC USA; 17 The Institute for Translational Genomics and Population Sciences, Department of Pediatrics, The Lundquist Institute for Biomedical Innovation at Harbor-UCLA Medical Center, Torrance, CA USA; 18 Metabolomics Platform, Broad Institute of MIT and Harvard, Cambridge, MA USA; 19 Department of Physiology and Biophysics, University of Mississippi Medical Center, Jackson, MS USA; 20 Department of Statistics, Harvard University, Boston, MA USA; 21 Center for Public Health Genomics, University of Virginia, Charlottesville, VA USA; 22 Boston University’s and National Heart, Lung and Blood Institute’s Framingham Heart Study, Framingham, MA USA

**Keywords:** Genome-wide association studies, Metabolomics, Predictive markers

## Abstract

Circulating metabolite levels may reflect the state of the human organism in health and disease, however, the genetic architecture of metabolites is not fully understood. We have performed a whole-genome sequencing association analysis of both common and rare variants in up to 11,840 multi-ethnic participants from five studies with up to 1666 circulating metabolites. We have discovered 1985 novel variant-metabolite associations, and validated 761 locus-metabolite associations reported previously. Seventy-nine novel variant-metabolite associations have been replicated, including three genetic loci located on the X chromosome that have demonstrated its involvement in metabolic regulation. Gene-based analysis have provided further support for seven metabolite-replicated loci pairs and their biologically plausible genes. Among those novel replicated variant-metabolite pairs, follow-up analyses have revealed that 26 metabolites have colocalized with 21 tissues, seven metabolite-disease outcome associations have been putatively causal, and 7 metabolites might be regulated by plasma protein levels. Our results have depicted the genetic contribution to circulating metabolite levels, providing additional insights into understanding human disease.

## Abstract (web summary)

Here, the authors perform a whole-genome sequencing association analysis of genetic variants in ≤11,840 multi-ethnic participants with ≤1666 circulating metabolites, discovering 1985 novel variant-metabolite associations and insights into human disease.

## Introduction

Circulating metabolite levels are highly heritable^1^, and positioned along the pathway between the genetic determinants and a wide variety of health outcomes. The latter include numerous Mendelian disorders, in which imbalanced blood or tissue metabolites levels are observed^2–5^, as well as various complex diseases, for which metabolite patterns are being investigated^6–9^. Most previous genetic studies of the human metabolome have focused on common variant analysis in European populations, predominantly using genome-wide association studies^10–15^, with few studies investigating Hispanic^16^ and African-American^17^ participants. Inclusion of ethnically diverse populations may lead to genetic discovery in broader populations, and therefore, better understanding of disease^18^. Additionally, most previous studies focused on investigating autosomal chromosomes. Exploration of the X chromosome can further enrich our understanding of the genetic architecture of metabolites. Adding to the complexity, the number of measurable circulating metabolites has been growing^19^, while only a modest proportion of the metabolites, typically including several hundreds of traits^20^, have been explored in relation to genotypic data.

In this investigation, we performed association analyses using whole-genome sequencing (WGS) to investigate the association of common and rare variants with 1666 circulating metabolites in multi-ethnic populations, using single variant and gene-centric analyses. We aggregated up to 11,840 adult participants of African, European, and Hispanic ancestries from five studies involved in the Trans-Omics for Precision Medicine (TOPMed) program for discovery analyses (full list of TOPMed authors is available in Supplementary Data 1)^1,21,22^. Our novel findings were further investigated using independent adult samples from TOPMed (up to 6763 participants), and two publicly available datasets (up to 11,322 participants), as replication. We also performed a gene network analysis, in which we integrated genome-wide associations with the human protein interactome to discover important interactions among metabolite-associated genes and their functions in biological pathways.

The centralized analyses utilizing jointly called WGS and harmonized metabolite data enable us to rapidly detect common and rare variants with maximized statistical power. The associations discovered in the present investigation advance our knowledge on the genetic architecture of circulating metabolites, as well as provide context for the identification of further connections between metabolic processes and disease phenotypes.

## Results

### Study design

**Fig. 1:**
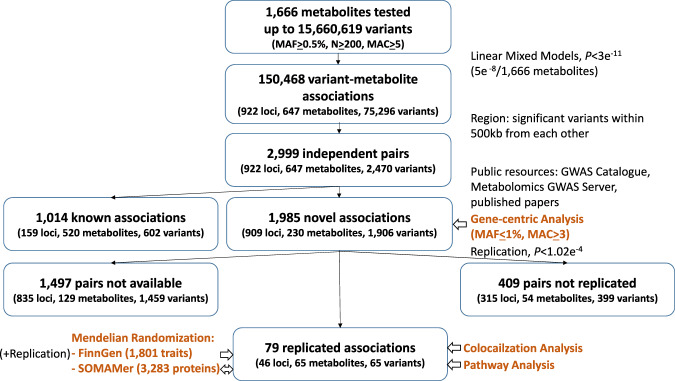
Study design. We performed single variant analysis of up to up to 15,660,619 variants with each of 1666 metabolites in up to 11,840 participants (Methods). Summary association statistics for variants in novel loci with *P*-value ≤ 3 × 10^−11^ (Methods section and Supplementary Data 5) were obtained from five independent studies (up to 18,085 participants). Only variants that were associated with a metabolite at *P*-value ≤ 1.02 × 10^−4^ in the replication analyses and had concordant directions of effect across studies (“Methods” section) were considered replicated.

In the discovery analyses we analyzed up to 15,660,619 common (Minor Allele Frequency [MAF] ≥5%), low-frequency (1% < MAF < 5%) and rare (MAF ≤ 1%) variants belonging to autosomal chromosomes and the X chromosome for association with 1666 rank-normalized circulating metabolites in up to 11,840 participants (mean age at 56.7 years old, 57% women) from a pooled sample of 1843 African-American (AA), 5938 European American (EA), and 4059 Hispanic (HIS) participants from the Atherosclerosis Risk in Communities study (ARIC), Hispanic Community Health Study/Study of Latinos (HCHS/SOL), Framingham Heart Study (FHS), Cardiovascular Health Study (CHS), and Multi-Ethnic Study of Atherosclerosis (MESA) (Methods). For replication analysis, we obtained summary statistics from up to five cohort studies (independent participants from FHS, Women’s Health Initiative [WHI], Jackson Heart Study [JHS], FENLAND, TwinsUK), including 2466 AA and 15,619 individuals of European ancestry, for a total sample size of up to 18,085 individuals (“Methods” section). The information on participating cohorts, as well as metabolite measurement methods and genotyping information is presented in Supplementary Data 2. Demographics of study participants, the biochemical name, pathway and missingness for each metabolite are summarized in Supplementary Data 3. The study design, applied statistical and functional analyses, and an overview of the known and novel findings are displayed in Fig. 1.

### Single Variant Tests

Overall, 150,468 single variant-metabolite associations reached the statistical significance threshold (*P*-value ≤ 3 × 10^−11^); 2999 associations were conditionally independent (*P*-value_conditional_ ≤ 5 × 10^−8^, “Methods” section), of which 1014 pairs (602 variants, 520 metabolites, 159 loci) were known (Supplementary Data 4) and 1985 pairs (1906 variants, 230 metabolites, 909 loci) were novel (with 708 loci reported for the first time for any metabolite, Supplementary Data 5). Inflation in our whole genome-wide single variant tests was well controlled with mean of genomic control lambda at 1.00 (standard deviation [SD] = 0.03, Supplementary Data 3). Consistent with our previous report^16^, rare and low-frequency variants (0.5% ≤ MAF < 5%) on average had 6.2 times larger effect on metabolites levels compared to common variants. The mean effect was at 1.39 SD and 0.22 SD change per minor allele for rare and low-frequency, and common variants respectively (Supplementary Fig. 1). Likewise, around 63% of detected variants belonged to genes, harboring 9% exonic variants (Supplementary Fig. 1)^16^.

Among 1985 novel independent associations identified, 488 statistically significant variant-metabolite association pairs were available for replication, and 79 pairs of 65 unique variants and 65 metabolites were successfully replicated (*P*-value ≤ 1.02 × 10^−4^, with consistent direction of effect in both discovery and replication sets), with explained variances ranging from 0.3% to 17% (Supplementary Data 6). Novel replicated loci affect metabolites from eight super pathways, including lipid-related metabolites (46%) amino acids (30%), cofactors and vitamins (9%), nucleotides (9%), carbohydrates (4%), organic acids (4%), energy (2%), and xenobiotics (2%), with 13 loci affecting more than one metabolite. Overall, among 79 novel replicated findings, the signals are relatively consistent across three ancestries − 73 had the same direction of effect across all the analyzed ancestries (Supplementary Data 5c). We attempted to extend those novel variant-metabolite pairs into two pediatric studies (1734 Hispanic children, “Methods” section), however, only one association was validated (rs7458962 - methylated nucleoside 5-methyluridine pair, (Supplementary Data 7), suggesting limited generalizability, which may be in part due to the focus of Hispanic background and asthma condition for the pediatric populations.

Genetic loci associated with metabolites identified on the X chromosome are sparse. We identified 18 novel loci on the X chromosome, 3 of which were successfully replicated. For example, the strongest associations for the novel replicated loci were detected on the chromosome X, for two amino acid-related metabolites involved in lysine metabolism (N-6-trimethyllysine - *TMLHE*, *P*-value = 9.89 × 10^−68^; N6-acetyllysine - *HDAC6*, *P*-value = 9.27 × 10^−57^). *TMLHE* encodes trimethyllysine dioxygenase, which converts trimethyllysine into hydroxytrimethyllysine in the carnitine biosynthesis pathway. *HDAC6* encodes histone deacetylase 6, a protein implicated in deacetylation of lysine residues on the N-terminal part of the core histones^23^. The minor A allele of the missense variant, rs61735967 (MAF = 2.1%), in *HDAC6*, was associated with high levels of N6-acetyllysine, a risk factor for neurological deficits.

### Gene-based rare variant analysis

To explore the effect of an aggregation of rare variants in each of 17,174 genes for 230 metabolites associated with novel loci (Fig. 1), we performed gene-centric analysis using STAAR-O, a newly developed method that provides powerful and robust rare variant association tests by dynamically incorporating multiple functional annotations (“Methods” section)^24,25^. STAAR-O groups rare variants into multiple coding and non-coding masks for each gene, including putative loss of function ([pLOF], stop gain, stop loss and splice), missense, synonymous, promoter and enhancer masks.

We detected 253 statistically significant (*P*-value ≤ 1.05 × 10^−9^, accounting for 17,174 analyzed genes, 230 metabolites and 12 categories) metabolite-gene-functional category associations. A total of 128 metabolite-gene association pairs (including 75 coding and 73 non-coding genes), had 106 unique genes associated with 45 metabolites (Supplementary Data 8 and 9 and Supplementary Fig. 2a). Thirty-nine identified gene-metabolite pairs (58 gene-metabolite-functional category associations) are located outside of novel or known loci identified using single variant analysis; 78 gene-metabolite pairs were located within known loci, and 11 – within novel loci (including 8 replicated locus-metabolite associations). Three replicated variant-metabolite pairs were annotated to genes that were also statistically significant in gene-centric analyses with respective metabolites (guanidinoacetate - *SLC25A45*, deoxycarnitine - *SLC25A45*, and N-acetylputrescine - *HDAC10*).

Gene-centric analysis implicated a biologically plausible gene *ALPL*, located ~55 Mb downstream of rs1697421, aggregation of missense variants in which was significantly associated (*P*-value = 3.87 × 10^−10^) with glycerol 3-phosphate levels. Interestingly, *ALPL* encodes the tissue-nonspecific alkaline phosphatase protein – an enzyme involved in the dephosphorylation of several phosphorus-containing metabolites. Mutations in this gene have been linked to hypophosphatasia^26,27^, a disorder characterized by loss of mineralization and joint pain.

### Co-localization of metabolites with eQTLs

To interpret the underlying biological activity beyond the identified replicated loci, we performed colocalization analysis with gene expression in GTEx V8 to investigate whether any of the 46 metabolite loci containing replicated variant-metabolite pairs also have effect on gene expression levels in various tissues (Supplementary Data 10). We identified that across 25 genetic loci, 29 variants (40 variant-metabolite pairs), have evidence of colocalization with 40 tissues (posterior probability, PPr > 0.6). For the majority of these loci (18 loci, 26 locus-metabolite associations), a single potential causal variant underlies both the expression of a single gene and the metabolite(s).

**Fig. 2:**
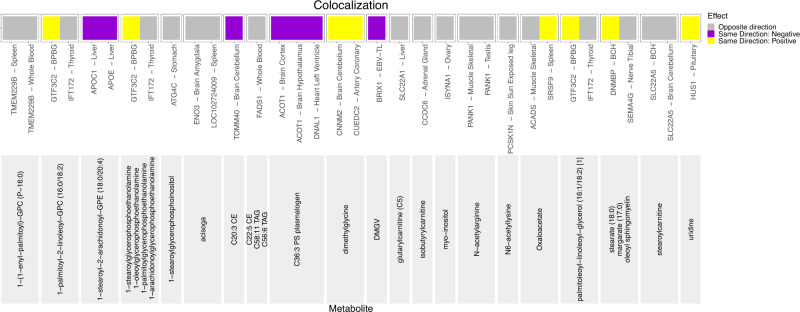
Novel replicated variant-metabolite pairs colocalizing for the association with gene eQTLs. The direction of the effect of minor allele on metabolite levels and gene expression is shown in the legend. At the bottom of the graph, in light gray, are the names of the metabolites. Above the names of metabolites are eQTL gene-tissue pairs. If both the effect of minor allele on metabolite levels and on gene expression is more than 0, such variant-metabolite-gene eQTL combinations are marked in yellow, and annotated as “Same Direction: Positive”. If the effect of minor allele on metabolite levels and on gene expression is less than zero, such variant-metabolite-gene eQTL combinations are marked in purple, and annotated as “Same Direction: Negative”. If the effect of minor allele on metabolite levels is less than zero and the effect of minor allele on gene expression is more than zero, or vice versa, such variant-metabolite-eQTL combinations are marked in gray, and annotated as “Opposite Direction”. Additionally, the following acronyms were used for tissues: BPBG brain putamen basal ganglia, BCH brain cerebellar hemisphere, EBV-TL - Cells EBV-transformed lymphocytes.

Overall, nineteen replicated novel independent variants were colocalizing for the association with 26 metabolites and 26 gene eQTLs in 21 tissues (Fig. 2), suggesting that the expression of these genes may be the reason behind the variation of metabolite levels associated with these loci. Among 26 novel replicated variant-gene eQTL pairs, 27% were pertaining to 5 missense variants, and 13 - to 10 intronic variants. Additionally, four intronic variants (rs67481496-*ETFDH*, rs17125278-*PANK1*, rs1077989-*TMEM229B* and chromosome 6:160139865-*SLC22A1*), one synonymous variant (rs10405636-*SSBP4*) and one missense variant (rs1799958-*ACADS*) colocalize with expression of the gene to which they were annotated (with the most deleterious functional consequence).

### Gene network and pathway analysis

To enhance the biological insight obtained from our findings, we performed gene network analysis and pathway enrichment analysis of the 65 metabolites with statistically significant replicated findings, aiming to identify gene networks associated with each metabolite. The dense module search of GWAS (dmGWAS version 2.7) was used for our network-based analysis, where the input was gene-level weights, based on MAGMA association scores, and the human protein interactome, comprised of experimentally validated protein-protein interactions (PPIs), annotated in PathwayCommons^28^. We then performed enrichment analysis for gene sets from the top resulting network modules using the over-representation analyses (ORA, see Methods).

Overall, 31 metabolite-gene set pair had significant association (*P*-value ≤ 2.70 × 10^−8^ Bonferroni correction for 28,438 Biological Process terms in Gene Ontology annotations and 65 metabolites). Among those, 2, 6, and 4 metabolite-gene set pairs contained genes belonging to 2 known metabolite loci, 4 novel metabolite loci, and 3 novel replicated metabolite loci respectively. The latter included one metabolite-gene pair (*N*-acetylputrescine*-HDAC10*, Supplementary Data 11), which was also detected and replicated in single variant analysis (missense variant rs61748567 - *N*-acetylputrescine) and detected by the coding gene-centric analysis. Among them, several genes associated with *N*-acetylputrescine, were enriched in several GO Biological Process terms, including intracellular receptor signaling pathway (GO:0030522) and covalent chromatin modification (GO:0016569), with *HDAC10* being a part of the latter pathway. HDAC10 is involved in deacetylation of polyamines, including *N*-acetylputrescine^29^, whereas other members of the HDAC family have been shown to act as deacylases as well as deacetylases^30^. Moreover, putrescine (*N*-acetylputrescine precursor) depletion was previously suggested to affect chromatin structure in brain tumor cells^31^. Therefore, our data provide additional biological mechanisms of the genetic and metabolomic engagement in the above processes.

### Mendelian randomization

To identify putatively causal relationships between various phenotypes in FinnGen and the 65 metabolites associated with replicated variants, we performed a series of MR analyses (Methods) using: (1) 1801 phenotypic traits from FinnGen, and (2) summary statistics for 3283 plasma proteins to elucidate the possible causal pathways (see Methods).

**Fig. 3:**
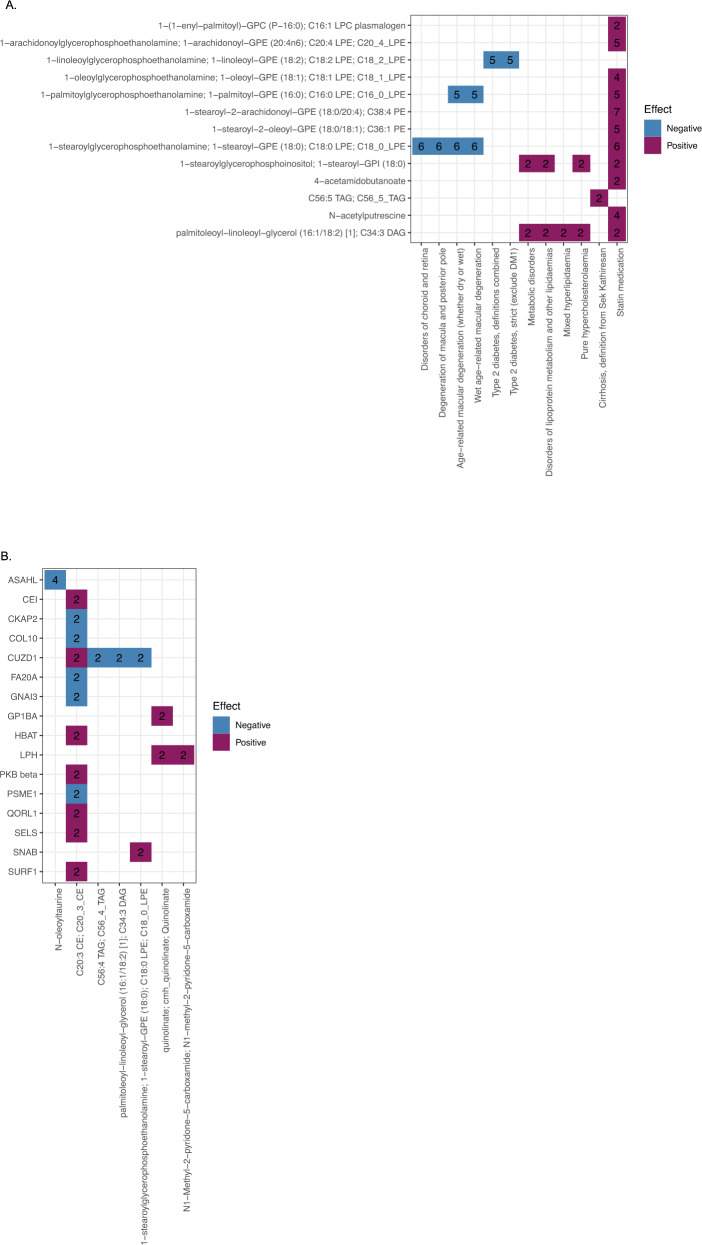
MR results. **A.** Metabolites effect on FinnGen phenotype traits. FinnGen phenotype traits are provided on the *x*-axis, metabolites are provided on the *y*-axis. The color indicates whether increase in metabolite level increases FinnGen phenotype odds (purple), or decreases FinnGen phenotype odds (blue). The number on the center of each square indicates the number of variants used to obtain each result. **B.** pQTL effect on metabolites. Metabolites are provided on the x-axis, pQTL are provided on the y-axis. The color indicates whether increase in pQTL levels increases metabolite levels (purple), or decreases metabolite levels (blue). The number on the center of each square indicates the number of variants used to obtain each result.

Using summary statistics from FinnGen, 27 statistically significant (*P*-value ≤ 1.51 × 10^−7^, accounting for 65 metabolites, 1801 traits and 3283 pQTLs) metabolite-outcome association pairs were detected, where 12 (5 metabolites, 6 FinnGen outcomes) had two Instrumental Variables (IV) available (Supplementary Data 12 and Fig. 3). Among 15 associations with more than two instrumental variables (9 metabolites, 7 FinnGen outcomes), seven associations (3 metabolites, 6 outcomes) remained nominally significant (*P*-value < 0.05) in MRPRESSO outlier test. For example, genetically regulated higher 1-linoleoylglycerophosphoethanolamine levels demonstrate a putative causal effect on Type 2 Diabetes (OR [95%CI] = 0.82 [0.80–0.85]). Additionally, higher 1-stearoylglycerophosphoethanolamine levels show putative causal effects on disorders of choroid and retina (OR [95%CI] = 0.81 [0.78–0.84]), degeneration of macula and posterior pole (OR [95%CI] = 0.71 [0.67–0.76]), wet age-related macular degeneration (AMD, OR [95%CI] = 0.49 [0.44–0.55]), and age-related macular degeneration (OR [95%CI] = 0.56 [0.51–0.61]). Likewise, higher 1-palmitoylglycerophosphoethanolamine (GPE) levels have a significant causal effect on age-related macular degeneration (OR [95%CI] = 0.60 [0.55–0.66]). The latter two associations are due to several conditionally independent variants in or near *ALDH1A2* and *GCKR*, associated with 1-palmitoyl-GPE and 1-stearoyl-GPE levels. *ALDH1A2* is involved in retinoic acid synthesis^32^, and is known to be associated with AMD^33^. *GCKR* is a well-known gene associated with diabetes, and diabetes is a risk factor for AMD^34^. Wet AMD is accompanied by severe loss of photoreceptors and ganglion cells^35^. Metabolite 1-palmitoyl-GPE was reported to induce neurite outgrowth;^36^ therefore, 1-palmitoyl-GPE may play a protective role against the loss of ganglion cells in wet AMD. Moreover, 1-palmitoyl-GPE and 1-stearoyl-GPE both belong to saturated lysophosphatidylethanolamine species. Given the concordant OR, it is possible that saturated lysophosphatidylethanolamines in general might influence the age-related macular degeneration. However, functional investigations are needed to support these findings.

To determine robustness of the identified metabolite–phenotype putatively causal associations, we performed additional MR analyses using a set of independent studies (UKBiobank, European Bioinformatic Institute [EBI] and BioBank Japan [BBJ]), matched by the outcome. Seven metabolite-outcome association pairs met stringent Bonferroni correction (*P*-value ≤ 0.05/11 = 4.55 × 10^−3^) and had the same direction of effect as in metabolite FinnGen MR analyses (Supplementary Data 12).

The interaction between metabolite and protein plays a critical role in controlling cellular homeostasis^37^. To identify possible causal pathways, we performed MR to identify putatively causal relationships between plasma proteins using summary statistics for 3283 protein quantitative trait loci (pQTLs) from the INTERVAL study^38^ and 65 metabolites associated with the replicated genetic loci. We detected 52 statistically significant pairs (*P*-value_IVW MR_ < 4.93 × 10^−7^, accounting for 65 metabolites and 1561 pQTLs), where twelve metabolites were affected by 44 proteins (Supplementary Data 13 and Fig. 3b). For example, genetically regulated increased *N*-acylethanolamine-hydrolyzing acid amidase (ASAHL) levels were causal of decreased *N*-oleoyltaurine levels. ASAHL plays role in *N*-acyl ethanolamines degradation^39–43^, and has hydrolytic activity against the ceramides^39^. Therefore, our data suggests that ASAHL may also affect *N*-acyl amines. Metabolites may reversely affect protein levels, such as protein-metabolite interactions or post-translational modifications^44,45^. We additionally tested the potential causal associations between metabolite and plasma proteins using the same analytical approach. There were eleven metabolites causally associated with 17 proteins with a total of 24 significant metabolite-pQTL associations pairs (with *P*-value_IVW MR_ < 1.51 × 10^−7^, accounting for 65 metabolites, 1801 traints and 3283 pQTLs). For example, 1-palmitoyl-glycerophosphoethanolamine (GPE) levels were predictive of P5I11 levels, and 1-stearoyl-GPE levels - predictive of GGT2, P5I11, PSG5, and PKB beta levels (product of *AKT2*); Supplementary Data 14 and Supplementary Fig. 4.

## Discussion

We conducted a WGS study to detect genetic loci associated with 1666 circulating metabolites in a multi-ethnic population, and identified 75 novel replicated metabolite-genetic locus associations, with 22 associations driven by nonsynonymous variants. A comprehensive gene-centric rare variant analysis was performed for a subset of metabolites, with 126 gene-metabolite pairs detected, showing associations between 45 metabolites and 105 genes. Using Mendelian Randomization, we showed that the levels of 13 metabolites were associated with the risk of 12 phenotype outcomes, including type 2 diabetes and macular degeneration. Moreover, 16 metabolites were associated with 29 protein QTLs. Our study represents the first WGS of human metabolome in multi-ethnic population, which provides novel insights beyond previous GWAS.

Previous metabolite genetic studies often restricted to single metabolomic platform^1,46^, used other platform results for replication^46^, or combined cross-platform results using meta-analyses^21^. We demonstrated a contemporary approach to analyze cross-platform harmonized metabolite levels in pooled samples, which largely improved computational efficiency. Our results showed that this new approach well controlled genomic inflation, reproduced hundreds of known metabolite loci, and enabled novel gene identification via rare variant analyses within a modest sample set, revealing the advantage of joint analyses, specifically for large genomic initiatives, where multiple studies are involved. Importantly, we are the first to extend metabolite genetic association discovery into multi-ethnic populations. The diverse ancestral background promoted novel findings beyond studies focusing on single ancestral population^1,16,47^, even with tens of thousands of participants^21^. Furthermore, our findings provided additional insights into biological pathways by investigating the interacting effects between proteins and metabolites, where most past studies dedicated to illustrate the putative causal effect between metabolites and health outcomes.

Although using pooled samples is considered computationally efficient, the variant-set test is intensive and costly for whole-genome analyses. We performed gene-centric rare variant analyses among 230 metabolites, which had significant common variant findings, as we considered those metabolites had relatively high heritability and used this opportunity to explore rare variants contribution to those metabolites. For the current analyses, more than 5500 jobs were run, including both single variant and gene-centered analyses, on the DNAnexus platform using the instance type “mem3_ssd1_v2_x32”, which provides 32 cores, 224GB of memory (> =7GB/core), and 640GB of solid-state drive storage (20GB/core)^48^. Future efforts are warranted to further explore rare-variant effects across all metabolites.

To understand possible mechanisms underlying the replicated novel findings, we applied versatile analytic approaches, including colocalization and pathway analyses, which provided an additional level of detail for the identified loci. Using colocalization analyses, we identified 18 unique loci where the novel replicated variant colocalized with the eQTL for 26 unique genes in GTEx tissues, highlighting the biologically plausible genes. For example, the splice site intronic *SLC22A1* variant (chromosome 6:160139865) is associated with increased levels of the lysine metabolism metabolite glutarylcarnitine (C5). Our colocalization analysis also showed that 6:160139865 colocalizes with decreased SLC22A1 levels in the liver - the primary expression site of the latter protein^49^. *SLC22A1* encodes a plasma membrane transporter organic cation transporter 1 (OCT1)^49^, which plays a role in regulating levels of acylcarnitines^50^ and isobutyrylcarnitine^51^. Therefore, our data suggests that *SLC22A1* plays role in regulation of blood levels of glutarylcarnitine.

Another replicated intronic *ELL* variant rs8109573 is colocalizing with decreased expression of ISYNA1 in ovaries and increased myoinositol levels. *ISYNA1* is located ~68 kilo base-pairs (kbp) downstream of *ELL*, and encodes an inositol-3-phosphate synthase enzyme, which plays a key role in myoinositol synthesis pathway^52^. Intronic variant, rs67481496, located in *ETFDH* and ~7 kbp from the replicated variant rs17843966, is colocalizing with increased expression of ETFDH in heart tissues, liver, and skeletal muscle and decreased glutarylcarnitine (C5-DC) levels. The latter association is consistent with the association observed in patients with glutaric acidemia, caused by deleterious mutations in the *ETFDH* gene, which, among other metabolite changes, is accompanied by increased blood levels of glutarylcarnitine (C5-DC)^53,54^.

Pathway analysis provided additional biological insights. For example, top results of the pathway analysis identified biological functional terms of coagulation and the regulation of body fluid levels for the metabolite acylcarnitine linoleoylcarnitine (C18:2 carnitine), with 15 overlapping genes between these pathway annotations (Supplementary Data 11 and Supplementary Fig. 3). None of the genes in the gene sets for those pathways belonged to ‘known’ or ‘novel’ loci identified by single variant analysis for this metabolite, and none were statistically significantly associated with linoleoylcarnitine in gene-centric analysis. Nevertheless, gene network analysis of the GWAS summary statistics allowed us to identify these important gene interactions. Previously, observational studies showed the involvement of acylcarnitines in blood coagulation. For example, Deguchi et al. showed that long-chain acylcarnitines, including linoleoylcarnitine, are lower in patients with venous thromboembolism, compared to age-matched controls (*P*-value = 0.02), and that linoleoylcarnitine possesses anticoagulant properties, possibly, due to the capability of acylcarnitines to bind with factor Xa^55^. Later, Zeleznik et al. showed that metabolites in the acylcarnitine pathway, including linoleoylcarnitine, are depleted in the intermediate/high-risk group of pulmonary embolism compared to the low-risk group^56^, further supporting the notion of acylcarnitines involvement in coagulation. However, no coagulation-related genetic loci were previously identified for linoleoylcarnitine, making our pathway analysis the first genetic evidence to link this metabolite to blood coagulation.

The MR analyses have been adopted to various phenotypes to help identify the causal relationships, specifically the potential bi-directional associations between proteins and metabolites. For example, genetically regulated higher levels of CUZD1 contribute to decreased levels of several lipid-related metabolites, including C56:4 TAG, C34:3 DAG, C20:3 CE, and 1-stearoyl-GPE. CUZD1 is located in the secretory granules in the pancreas and pancreatic secretions. Although it’s exact biological function is unknown, it is thought to play a role in immune response due to its involvement in inflammatory bowel disease (IBD)^57^, with anti-CUZD1 autoantibodies suggested as a marker of the IBDs^58^. At the same time, patients with IBDs may have altered lipid profiles, compared to healthy individuals^59^. Therefore, our data suggests that CUZD1 affects the levels of various lipid-related metabolites, although further functional studies are needed to explore these relationships further.

Likewise, genetically high levels of *N*-acetylarginine decrease the levels of IGFBP-6. IGFBP-6 has cancer-protective properties, plays a role in the immune system^60,61^ and in neuronal protection^62^. *N*-acetylarginine is a guanidino compound that is capable of inducing seizures in animal experiments;^63^ high levels of this compound present in argininemia that is characterized by neurological symptoms^64^.

In this project, we applied a stringent Bonferroni correction to define significance for replication, and a modest amount (79 out of 488) of our novel association were replicated. However, most associations (304 out of 488) showed the same direction of effects, providing supportive evidence of our findings, which are warranted for further investigation. Of note, our replication set included participants from several studies with a modest sample size, which may impact the replication due to possible heterogeneity across studies and lack of sufficient statistical power, specifically for low-frequency variants.

Of note, most our novel findings are consistent across ancestries. For examples, associations of rs1697421- glycerol 3-phosphate, rs6440123 − 1-stearoyl-2-oleoyl-GPE (18:0/18:1), rs68008113 - cerotoylcarnitine (C26), rs113680823 - arabitol/xylitol and rs5112 − 1-stearoyl-2-arachidonoyl-GPE (18:0/20:4) had the same directions of effect in three ancestries, European, African and Hispanic Americans. High statistical significance (*p*-value < 1.85 × 10^−5^ in each ancestral group) in European and Hispanic Americans were observed though there was no significance in African Americans. Ancestry-specific results (Supplementary Data 5c) are provided to facilitate further dissection of potential differences across ancestral groups.

In summary, we showed the feasibility of performing computationally efficient pooled analysis, using both metabolomics and WGS data, which can be applied for the future research projects. Additionally, this study provides further determination of the genetic architecture of circulating metabolites in a multi-ethnic population, using both common and rare variants, comprehensive functional annotation, and a systematic identification of potential causal relationships between the genes, metabolites, various phenotypes and plasma protein levels. Our results can be widely used in future studies to expand further our understanding of the biological processes in health and disease.

## Methods

### Genetic studies

Five cohorts contributed to the discovery stage of the analysis (ARIC, FHS, CHS, MESA, and HCHS/SOL) with a total of 11,840 participants, including 5938 EA, 1843 AA, and 4059 HIS. Study-specific characteristics, metabolite measurement procedures, genetic sequencing information, and quality control for the studies are provided in Supplementary Data 2A, while basic study characteristics are listed in Supplementary Data 3.

For replication, summary association statistics were requested (for the novel variant-metabolite associations with *P* < 3 × 10^−11^) from three cohorts: FHS (2969 EAs), WHI (1328 EA), and JHS (2466 AAs). Additionally, we obtained publicly available summary statistics from 9363 European FENLAND participants^21^ and 1959 EUR TwinsUK participants^1^. In total, up to 18,085 individuals (including 15,619 European ancestry participants and 2466 AA participants) were available for replication meta-analysis. Study-specific characteristics are provided in Supplementary Data 2B.

All the participating studies were approved by corresponding institutional review boards, and all participants provided written informed consent.

### Metabolite measurements

Details of the metabolites measurements are provided in Supplementary Data 2, while for the previously published studies, these can be found in the respective manuscripts^1,21^. In brief, blood samples were collected in participating studies, processed and stored at −70 °C since collection. Overall, 1666 metabolites were measured by untargeted, gas and/or liquid chromatography-mass spectrometry-based quantification protocol (Supplementary Data 3)^65,66^. In HCHS/SOL and ARIC, metabolites were measured by Metabolon Inc. (Durham, NC) platform. For CHS, FHS, JHS MESA, and WHI, metabolites were measured by Broad Institute.

### Genotyping, quality control, and imputation

Blood samples were sequenced on the Illumina HiSeq X; for MESA and FHS, sequencing was performed by the Broad Institute of MIT and Harvard; for CHS and HCHS/SOL - by the Baylor College of Medicine Human Genome Sequencing Center, while for ARIC - by both centers. Variants calling was completed using the GotCloud pipeline^67^.

Quality control procedures have been described elsewhere^67^. In short, variant filtering was performed by calculating Mendelian consistency scores using known familial relatedness and duplicates, and by training a Support Vector Machine classifier between known variant sites (positive labels - SNPs polymorphic either in the 1000 Genomes Omni2.5 array or in HapMap 3.3, with additional evidence of being polymorphic in the sequenced samples) and Mendelian inconsistent variants (negative labels - having the Bayes Factor for Mendelian consistency <0.001; or if 10% or more of families or pairs of duplicate samples show Mendelian inconsistency within families or genotype discordance between duplicate samples). Additionally, excess heterozygosity filter was applied to variants with the Hardy–Weinberg disequilibrium *P*-value < 1 × 10^−6^ in the direction of excess heterozygosity after accounting for population structure. Mendelian discordance filter was applied when ≥5% of families show Mendelian inconsistency or genotype discordance.

### Statistical analysis

#### Single variant tests

We applied a two-stage procedure for rank normalization in genotype-metabolite association analyses^68^. The fully adjusted two‐stage approach was chosen due to its ability to reduce excess Type I errors and to improve statistical power, as well as due to having a lower degree of inflation compared to approaches without rank-normalization^68^. It has been widely applied to large-scale GWAS studies for complex traits^69–71^, including metabolomic measures in the mixed populations^16^. As in above studies, data preparation for the single variant analysis involved several steps. First, each of the 1666 metabolites were inverse rank normal transformed by study, race and batch. Second, we obtained the residuals using generalized linear mixed model adjusting for age, sex, race, study, and study variables (such as recruitment center), and the first 11 principal components with random effects accounting for inter-individual correlation (due to either relatedness, shared household, or census block group). Third, above residuals were inverse normal transformed, and finally, these inverse transformed residuals were used in the genetic analyses again adjusting for all of the aforementioned covariates, along with estimated glomerular filtration rate (eGFR)^72^. For this study, we considered both autosomal and X chromosome variants. Overall, up to 15,660,619 variants (MAF ≥ 0.5%, *N* ≥ 200, minor allele count [MAC] ≥ 5) were analyzed with each metabolite. Analyses were performed in GENESIS^73^, using additive genetic models. Significance for single variant analysis was defined as two-sided *P*-value ≤ 3 × 10^−11^ (accounting for ~1,000,000 independent variants and 1666 metabolites).

### Conditional analysis

Across the analyzed genome, we defined metabolite-associated genetic loci as containing all statistically significant variants within 500 kbp from each other. To account for linkage disequilibrium, we added 500 kbp to each side of the region, and all the overlapping regions were merged. We identified 922 loci, containing 2614 locus-metabolite pairs.

For every locus-metabolite pair, we performed conditional analysis using GENESIS to identify the independent leading variants. Conditioning was performed step-wise. In each round, conditioning was performed on the variant(s) with the lowest *P*-value in the region. Variants that were both statistically significant in the primary analysis (*P*-value ≤ 3 × 10^−11^) and genome-wide statistically significant (*P*-value_conditional_ < 5 × 10^−8^) in the conditional analysis were considered conditionally independent associations.

We identified 2999 conditionally independent variant-metabolite associations (Supplementary Data 4–5). A majority (2330) of the metabolite-genetic region pairs had one conditionally independent variant; 218 pairs had 2 conditionally independent variants; 41 pairs − 3; 18 pairs − 4; and 3 pairs − 6 conditionally independent variants. For each statistically significant independent variant-metabolite association, we used R to calculate proportion of variance in corresponding metabolite explained by the variant^74^.

### Annotation of the known and novel findings

To annotate the identified 2999 independent variant-metabolite associations, we obtained reports from the Metabolomic GWAS Server^12^, TwinsUK study^1^, GWAS Catalogue, GRASP Search, previous reports from our group^16,75–77^ and performed manual search through published papers to detect known loci that overlap with our findings. If a variant from a variant-metabolite pair was previously associated with any of the metabolites in its sub-pathway (Supplementary Data 3), the variant-metabolite pair was considered known, otherwise the variant-metabolite pair was considered novel.

### Replication analysis

We performed inverse-variance weighted meta-analysis of single variant summary statistics obtained from five studies (Supplementary Data 5), using meta version 4.18-0 R package. Out of 1985 novel variant–metabolite associations, 488 variant-metabolite associations (107 metabolites, 458 unique variants) were available in at least one replication cohort. Significant replication was defined as: (1) had two-sided *P*-value ≤ 1.02 × 10^−4^ = 0.05/488 in meta-analysis or in a single replication cohort (when association was available only in one cohort) and (2) had consistent direction of effect in both discovery and replication meta-analysis.

### Generalization in pediatric populations

We requested summary statistics for 1985 novel variant-metabolite associations from two children studies - Childhood Asthma Management Program (CAMP) and Genetic Epidemiology of Asthma in Costa Rica (CRA) (Supplementary Data 7), and obtained summary statistics for 51 novel variant-metabolite pairs. For these associations, we also performed inverse-variance weighted meta-analysis between the two studies (CAMP and CRA). Significant associations were defined as: (1) had two-sided *P*-value ≤ 9.8 × 10^−4^ = 0.05/51 in meta-analysis and (2) had consistent direction of effect in both discovery and meta-analysis.

### Gene-centric rare variant analyses

To test whether rare variants in aggregate affect metabolite regulation, we performed gene-centric rare variant analyses for 230 metabolites associated with novel loci, using variant-set test for association using annotation information omnibus test (STAAR-O) in discovery dataset^24^, which boosts the power of rare variant association tests by incorporating multiple variant functional annotations. For each test, we included variants with MAF ≤ 1% (Supplementary Fig. 2b). To ensure the robustness of the results, gene-metabolite associations with the sample size of <5000 and cumulative MAC of <100 were excluded. Aggregation was based on each of the following five functional variant categories for the gene-centric coding genome - missense, synonymous, putative loss of function (stop gain, stop loss, and splice), disruptive missense, and combined putative loss of function and disruptive missense. For the gene-centric non-coding genome, aggregation was performed based on the following seven variant categories: downstream, enhancer variants overlaid with Cap Analysis of Gene Expression (CAGE) sites, promoter CAGE, enhancer variants overlaid with DNAse HyperSensitivity (DHS), promoter DHS, upstream and UTR^25^. Gene-metabolite associations with two-sided *P*-value ≤ 1.05 × 10^−9^ (accounting for 17,174 analyzed genes, 230 metabolites, and 12 categories), were considered significant.

### Co-localization of metabolites with eQTLs

We performed co-localization analysis with GTEx V8 eQTLs summary to investigate whether, for the 65 identified novel replicated genetic locus-metabolite associations, these genetic loci share candidate variants with the gene expression levels. Analysis was performed using HyPrColoc, which can identify subsets of traits colocalizing at distinct causal variants in the genomic locus. For each genetic locus (Supplementary Data 3c), all metabolites associated with variants within the locus with evidence of replication were analyzed simultaneously. Variants represented in both discovery dataset and all 49 tissues of GTEx V8 dataset were included^78,79^ (prior structure: $p$ = 0.0001, $\gamma$ = 0.98, Supplementary Data 10). As colocalizing, we considered variants with posterior probability (PPr) > 0.6 for colocalization between metabolite(s) with gene eQTLs in tissue(s). We also performed sensitivity analyses for the co-localization results (including the metabolites and eQTLs detected in the primary co-localization analysis), to address the causal configuration of priors (Supplementary Data 10b).

### Gene network and pathway analysis

Gene-level association scores were obtained for each of the 65 metabolites, based on respective single variant summary statistics. The gene-level association analysis was performed by applying the MAGMA (Multi-marker Analysis of GenoMic Annotation) tool version 1.09a. MAGMA maps SNPs to genes during the “annotation step”, and then performs SNP-wise mean for each gene to obtain gene-level *P*-values during the “gene analysis step”^80^. In order to perform this gene-level association test, a mixed population linkage disequilibrium (LD) reference panel from the 1000 Genomes Project for individuals of American ancestry was used as input, and the default MAGMA parameters were applied. MAGMA employs a multiple regression model to assess the additive effects of single variant associations, while accounting for LD patterns. The 1000 Genomes Project LD reference panel of American ancestry, which includes individuals of multiple ethnicities, was used because it has been previously recommended as an appropriate reference for investigations of a mixed population^81^. The P-values were then transferred to *Z*-scores via the inverse normal distribution function. The calculated *Z*-scores were used as gene weights in our network-based analysis of GWAS signals. The dense module search of GWAS (dmGWAS version 2.7) tool was used to identify gene networks associated with each metabolite^28^. The dmGWAS method uses GWAS-based gene-level scores and a reference protein-protein interaction (PPI) network to identify gene network modules associated with a phenotype of interest. In this case, the reference PPI used was a collection from PathwayCommons, representing the human protein interactome, which included 39,240 annotations of experimentally validated PPIs^82^. Next, gene sets from the top 10 ranking network modules were extracted for each metabolite. The pathway enrichment analysis was performed for each of these gene sets by over-representation analyses (ORA, Supplementary Data 11). The ORA was performed by using the WebGestalt R package version 2019 with the Gene Ontology Biological Process term annotations for genome protein-coding genes. Default parameters were applied for ORA methods^83^.

### Mendelian randomization

We performed a MR analysis using the summary statistics for 1801 traits in 135,638 participants from FinnGen (R3 - public release of 16 June 2020). For each of 65 metabolites associated with replicated variants, we used all conditionally independent variants. For each variant, we obtained a causal estimate as the ratio of the association of the variant with each of 1801 FinnGen traits.

To determine robustness of the identified statistically significant metabolite - FinnGen outcome phenotype associations, we performed additional MR analyses using our summary statistics as exposure and an additional set of independent studies (UKBiobank, EBI, and BBJ) as outcomes. Thirty-three statistically significant metabolite-FinnGen outcome association pairs were matched to a comparable outcome obtained from one of the above datasets (Supplementary Data 12).

We also used the Sun et al^38^. summary statistics for plasma proteins for metabolite-pQTL MR^38^. For above two MR analyses, associations with *P*-value_IVW MR_ < 1.51 × 10^−7^ (accounting for 65 metabolites, 1801 traits and 3283 pQTLs) were considered statistically significant.

We further performed MR for 1561 pQTL independent variants reported by Sun et al^38^. as IV, with each of 65 metabolites associated with replicated genetic loci as outcomes (significance threshold was set at *P*-value_IVW MR_ < 4.93 × 10^−7^, accounting for 65 metabolites and 1561 pQTLs).

For all of the above MR analyses, if exposure was associated with more than one variant, we performed a fixed effect inverse-variance weighting meta-analysis (IVW) using TwoSampleMR to obtain the overall estimates. Heterogeneity was assessed using the Q-statistic. Further, if the exposure was associated with more than two variants, we performed Egger MR using TwoSampleMR, as well as MR-PRESSO outlier test (to detect outlier IVs), using MR-PRESSO version 1.0^84^. Egger MR, although conservative, generates valid estimates even if not all the genetic instruments are valid, given that the Instrument Strength Independent of Direct Effect assumption holds^85^. Additionally, Egger MR intercept can help detect (unbalanced) pleiotropy. We obtained the F-statistic^86^ for the association of genetic variants with corresponding metabolites to assess instrument strength.

### Reporting summary

Further information on research design is available in the Nature Portfolio Reporting Summary linked to this article.

### Supplementary information

Supplementary Information

Description of Additional Supplementary Files

Supplementary Data 1

Supplementary Data 2-14

Reporting Summary

## Data Availability

Individual whole-genome sequence data from the TOPMed program are available through dbGaP. The dbGaP accession numbers are: Atherosclerosis Risk in Communities (ARIC) phs001211, Cardiovascular Health Study (CHS) phs001368, Framingham Heart Study (FHS) phs000974, Multi-Ethnic Study of Atherosclerosis (MESA) phs001416, and Hispanic Community Health Study - Study of Latinos (HCHS-SOL) phs001395. Data in dbGaP can be downloaded by controlled access with an approved application submitted through their website [https://www.ncbi.nlm.nih.gov/gap]. Individual metabolite data are available via request per each study policy. Summary statistics for single variant analysis of 1666 metabolites generated in this study are available at dbGAP Cohorts for Heart and Aging Research in Genomic Epidemiology (CHARGE) Consortium Summary Results from Genomic Studies, accession number phs000930.v10.p1 [https://www.ncbi.nlm.nih.gov/projects/gap/cgi-bin/study.cgi?study_id=phs000930.v10.p1] and dbGaP NHLBI TOPMed: Genomic Summary Results for the Trans-Omics for Precision Medicine Program, accession number phs001974.
