# Author Correction: Whole-Genome Sequencing Analysis of Human Metabolome in Multi-Ethnic Populations

**DOI:** 10.1038/s41467-023-42472-3

**Published:** 2023-10-19

**Authors:** Elena V. Feofanova, Michael R. Brown, Taryn Alkis, Astrid M. Manuel, Xihao Li, Usman A. Tahir, Zilin Li, Kevin M. Mendez, Rachel S. Kelly, Qibin Qi, Han Chen, Martin G. Larson, Rozenn N. Lemaitre, Alanna C. Morrison, Charles Grieser, Kari E. Wong, Robert E. Gerszten, Zhongming Zhao, Jessica Lasky-Su, NHLBI Trans-Omics for Precision Medicine (TOPMed), Honghuang Lin, Jeffrey Haessler, Jennifer A. Brody, Kari E. North, Kent D. Taylor, Clary B. Clish, James G. Wilson, Xihong Lin, Robert C. Kaplan, Charles Kooperberg, Bruce M. Psaty, Stephen S. Rich, Jerome I. Rotter, Ramachandran S. Vasan, Eric Boerwinkle, Bing Yu

**Affiliations:** 1 Human Genetics Center, School of Public Health, The University of Texas Health Science Center, Houston, TX USA; 2 Center for Precision Health, School of Biomedical Informatics, The University of Texas Health Science Center at Houston, Houston, TX USA; 3 Department of Biostatistics, Harvard T.H. Chan School of Public Health, Boston, MA USA; 4 Division of Cardiovascular Medicine, Beth Israel Deaconess Medical Center, Harvard Medical School, Boston, MA USA; 5 Department of Biostatistics and Health Data Science, Indiana University School of Medicine, Indianapolis, IN USA; 6 Channing Division of Network Medicine, Department of Medicine, Brigham and Women’s Hospital and Harvard Medical School, Boston, MA USA; 7 Retina Service, Massachusetts Eye and Ear, Harvard Medical School, 243 Charles Street, Boston, MA USA; 8 Department of Epidemiology and Population Health, Albert Einstein College of Medicine, Bronx, NY USA; 9 Department of Biostatistics, Boston University School of Public Health, Boston, MA USA; 10 Cardiovascular Health Research Unit, Departments of Medicine, Epidemiology, and Health Systems and Population Health, University of Washington, Seattle, WA USA; 11 Metabolon Inc., Morrisville, NC USA; 12 Division of Public Health Sciences, Fred Hutchinson Cancer Research Center, Seattle, WA USA; 13 Broad Institute of Harvard and MIT, Cambridge, MA USA; 14 Department of Medicine, University of Massachusetts Medical School, Worcester, MA USA; 15 Department of Epidemiology, University of North Carolina Gilling School of Global Public Health, Chapel Hill, NC USA; 16 Carolina Center of Genome Sciences, University of North Carolina, Chapel Hill, NC USA; 17 The Institute for Translational Genomics and Population Sciences, Department of Pediatrics, The Lundquist Institute for Biomedical Innovation at Harbor-UCLA Medical Center, Torrance, CA USA; 18 Metabolomics Platform, Broad Institute of MIT and Harvard, Cambridge, MA USA; 19 Department of Physiology and Biophysics, University of Mississippi Medical Center, Jackson, MS USA; 20 Department of Statistics, Harvard University, Boston, MA USA; 21 Center for Public Health Genomics, University of Virginia, Charlottesville, VA USA; 22 Boston University’s and National Heart, Lung and Blood Institute’s Framingham Heart Study, Framingham, MA USA

**Keywords:** Genome-wide association studies, Metabolomics, Predictive markers

Correction to: *Nature Communications* 10.1038/s41467-023-38800-2, published online 30 May2023

In this article, the author name Robert E. Gerszten was incorrectly written as Robert E. Gersztern. The original article has been corrected.

