# Supplementary material for: Whole-Genome Sequencing Analysis of Human Metabolome in Multi-Ethnic Populations
Source: Nat Commun. 2023 May 30;14:3111. doi: 10.1038/s41467-023-38800-2 (PMC10229598; doi:10.1038/s41467-023-38800-2)
Supplement: Supplementary file 1 — Supplementary Information [file 41467_2023_38800_MOESM1_ESM.pdf]

Whole-Genome Sequencing Analysis of Human Metabolome in Multi-Ethnic Populations

Supplementary Note 1

JHS and FHS

Acknowledgements

The Atherosclerosis Risk in Communities study has been funded in whole or in part with Federal funds from the National Heart, Lung, and Blood Institute, National Institutes of Health, Department of Health and Human Services (contract numbers HHSN268201700001I, HHSN268201700002I, HHSN268201700003I, HHSN268201700004I and HHSN268201700005I), R01HL087641, R01HL059367 and R01HL086694; National Human Genome Research Institute contract U01HG004402; and National Institutes of Health contract HHSN268200625226C. The authors thank the staff and participants of the ARIC study for their important contributions. Infrastructure was partly supported by Grant Number UL1RR025005, a component of the National Institutes of Health and NIH Roadmap for Medical Research. Metabolomics measurements were sponsored by the National Human Genome Research Institute (3U01HG004402-02S1).

The HCHS/SOL is a collaborative study supported by contracts from the National Heart, Lung, and Blood Institute (NHLBI) to the University of North Carolina (HHSN268201300001I / N01-HC-65233), University of Miami (HHSN268201300004I / N01-HC-65234), Albert Einstein College of Medicine (HHSN268201300002I / N01-HC-65235), University of Illinois at Chicago – HHSN268201300003I / N01-HC-65236 Northwestern Univ), and San Diego State University (HHSN268201300005I / N01-HC-65237). The following Institutes/Centers/Offices have contributed to the HCHS/SOL through a transfer of funds to the NHLBI: National Institute on

Minority Health and Health Disparities, National Institute on Deafness and Other Communication Disorders, National Institute of Dental and Craniofacial Research, National Institute of Diabetes and Digestive and Kidney Diseases, National Institute of Neurological Disorders and Stroke, NIH Institution-Office of Dietary Supplements. The Genetic Analysis Center at the University of Washington was supported by NHLBI and NIDCR contracts (HHSN268201300005C AM03 and MOD03). The authors thank the staff and participants of HCHS/SOL for their important contributions. Investigators website - http://www.cscc.unc.edu/hchs. Support for metabolomics data was graciously provided by the JLH Foundation (Houston, Texas).

Cardiovascular Health Study: This research was supported by contracts HHSN268201200036C, HHSN268200800007C, HHSN268201800001C, N01HC55222, N01HC85079, N01HC85080, N01HC85081, N01HC85082, N01HC85083, N01HC85086, 75N92021D00006, and grants R01HL128575, U01HL080295 and U01HL130114 from the National Heart, Lung, and Blood Institute (NHLBI), with additional contribution from the National Institute of Neurological Disorders and Stroke (NINDS). Additional support was provided by R01AG023629 from the National Institute on Aging (NIA). A full list of principal CHS investigators and institutions can be found at CHS-NHLBI.org. The content is solely the responsibility of the authors and does not necessarily represent the official views of the National Institutes of Health.

The WHI program is funded by the National Heart, Lung, and Blood Institute, National Institutes of Health, U.S. Department of Health and Human Services through contracts 75N92021D00001,

75N92021D00002, 75N92021D00003, 75N92021D00004, 75N92021D00005. The metabolic measurements for WHI were funded by grant X01-HL139376.

Multi-Ethnic Study of Atherosclerosis (MESA)" (phs001416.v1.p1) was performed at the Broad Institute of MIT and Harvard (3U54HG003067-13S1). Centralized read mapping and genotype calling, along with variant quality metrics and filtering were provided by the TOPMed Informatics Research Center (3R01HL-117626-02S1, contract HHSN268201800002I) (Broad RNA Seq, Proteomics HHSN268201600034I, UW RNA Seq HHSN268201600032I, USC DNA Methylation HHSN268201600034I, Broad Metabolomics HHSN268201600038I). Phenotype harmonization, data management, sample-identity QC, and general study coordination, were provided by the TOPMed Data Coordinating Center (3R01HL-120393; U01HL-120393; contract HHSN268180001I). Whole genome sequencing (WGS) for the Trans-Omics in Precision Medicine (TOPMed) program was supported by the National Heart, Lung and Blood Institute (NHLBI). WGS for "NHLBI TOPMed: Multi-Ethnic Study of Atherosclerosis (MESA)" (phs001416.v1.p1) was performed at the Broad Institute of MIT and Harvard (3U54HG003067-13S1). Core support including centralized genomic read mapping and genotype calling, along with variant quality metrics and filtering were provided by the TOPMed Informatics Research Center (3R01HL-117626-02S1; contract HHSN268201800002I). Core support including phenotype harmonization, data management, sample-identity QC, and general program coordination were provided by the TOPMed Data Coordinating Center (R01HL-120393; U01HL-120393; contract HHSN268201800001I). We gratefully acknowledge the studies and participants who provided biological samples and data for MESA and TOPMed. The MESA project is conducted and supported by the National Heart, Lung, and Blood Institute (NHLBI) in

collaboration with MESA investigators. Support for MESA is provided by contracts 75N92020D00001, HHSN268201500003I, N01-HC-95159, 75N92020D00005, N01-HC-95160, 75N92020D00002, N01-HC-95161, 75N92020D00003, N01-HC-95162, 75N92020D00006, N01-HC-95163, 75N92020D00004, N01-HC-95164, 75N92020D00007, N01-HC-95165, N01-HC-95166, N01-HC-95167, N01-HC-95168, N01-HC-95169, UL1-TR-000040, UL1-TR-001079, UL1-TR-001420, and NIDDK contract R01DK081572. Also supported in part by the National Center for Advancing Translational Sciences, CTSI grant UL1TR001881, and the National Institute of Diabetes and Digestive and Kidney Disease Diabetes Research Center (DRC) grant DK063491 to the Southern California Diabetes Endocrinology Research Center. Infrastructure for the CHARGE Consortium is supported in part by the National Heart, Lung, and Blood Institute (NHLBI) grant R01HL105756.

The Framingham Heart Study (FHS) acknowledges the support of contracts NO1-HC-25195, HHSN268201500001I and 75N92019D00031 from the National Heart, Lung and Blood Institute. We also thank the FHS study participants without whom this research would not be possible. Dr. Vasan is supported in part by the Evans Medical Foundation and the Jay and Louis Coffman Endowment from the Department of Medicine, Boston University School of Medicine.

The Jackson Heart Study (JHS) is supported and conducted in collaboration with Jackson State University (HHSN268201800013I), Tougaloo College (HHSN268201800014I), the Mississippi State Department of Health (HHSN268201800015I) and the University of Mississippi Medical Center (HHSN268201800010I, HHSN268201800011I and HHSN268201800012I) contracts

from the National Heart, Lung, and Blood Institute (NHLBI) and the National Institute on Minority Health and Health Disparities (NIMHD).

The authors would also like to thank Professor L. Adrienne Cupples (deceased) for her contributions to this manuscript.

A. B.

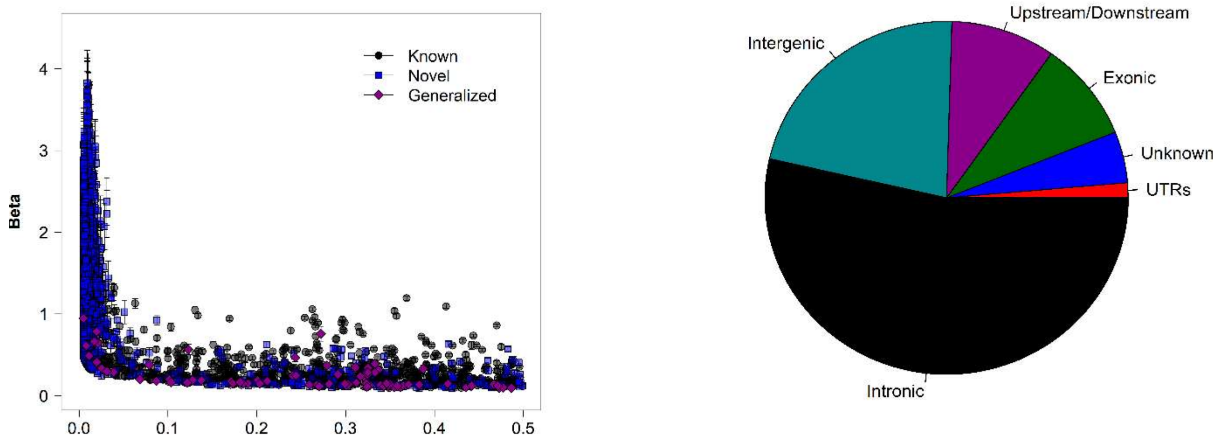

Supplementary Fig. 1. **A.** Minor allele frequency against absolute effect estimates for 2,999 variant-metabolite associations with standard errors. Blue squares are new metabolites-associated variants, dark grey dots represent variants at known loci and purple dots are metabolites-associated variants at novel loci. Data are presented as effect estimates +/- SE. All effect estimates and SEs are taken from the discovery analyses (up to 11,840 participants). B**.** Functional consequences of 2,470 unique variants associated with metabolites levels. Red colour is used for variants belonging to UTR regions, green – for exonic variants, purple – for upstream and downstream variants, teal – for intergenic variants, black – for intronic variants and blue – for variants not annotated to any of the above categories.

A.

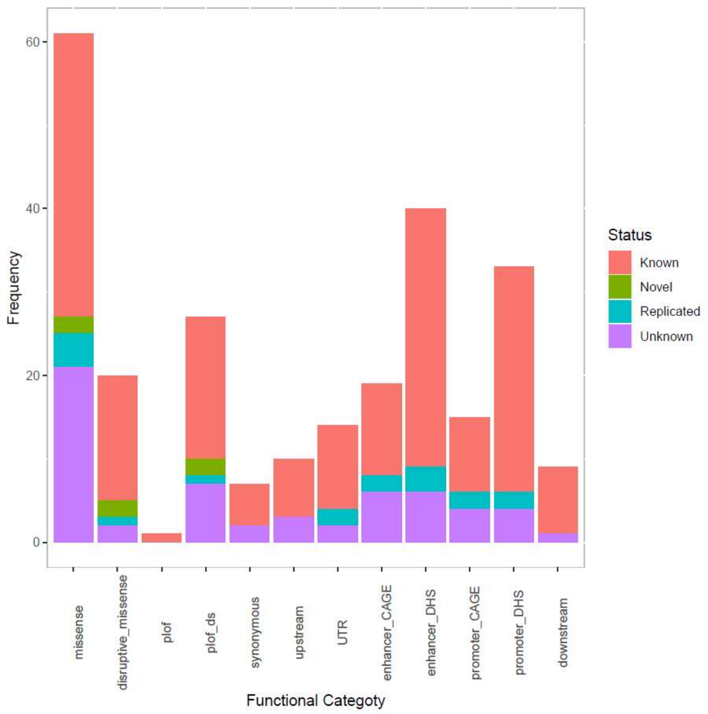

B.

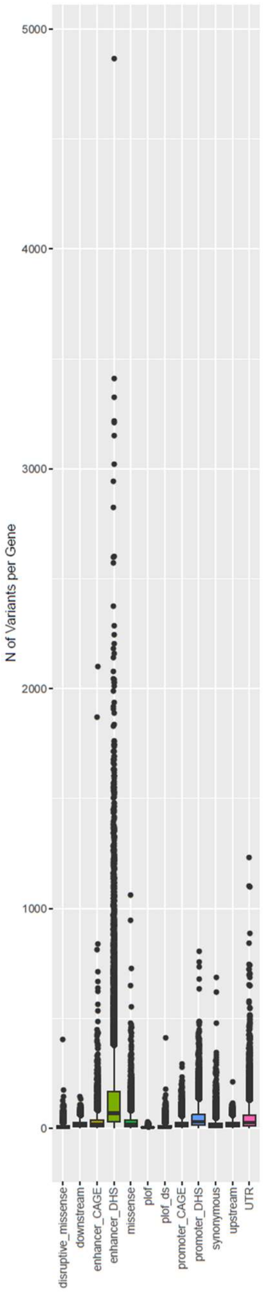

Supplementary Fig. 2. Gene-centric analysis results. A. Number of significant results by each of 12 functional categories, by the region-metabolite pair status, shown in the legend. The frequency of the significant results is shown on the y-axis. Each functional category is listed on the x-axis. The colours represent whether the genes belong to known (pink), novel (green), replicated (teal) loci as identified in the discovery analyses, or to loci not detected in the

discovery analyses (violet). B. Number of variants per gene per functional category for 230 metabolites. The center of each box denotes the median value. The upper and lower bounds of each box correspond to the 25th and 75th percentiles, respectively. The upper whisker extends from the upper box bound to the highest value that is within 1.5*IQR (inter-quartile range) of the upper box bound. The lower whisker extends from the lower box bound to the lowest value within 1.5*IQR of the lower box bound. Data beyond the end of the whiskers are plotted as points.

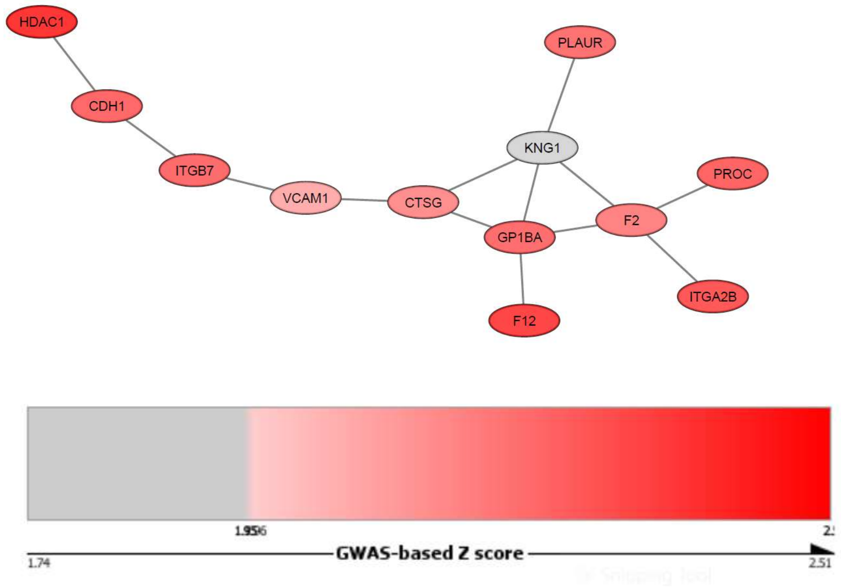

Supplementary Fig. 3. Gene network and pathway analysis. Top dmGWAS module of linoleoylcarnitine.

Each oval represents the gene within the module. The color of the oval corresponds to the GWAS-based Z score, and ranges from gray (low Z score) to red (high Z score).

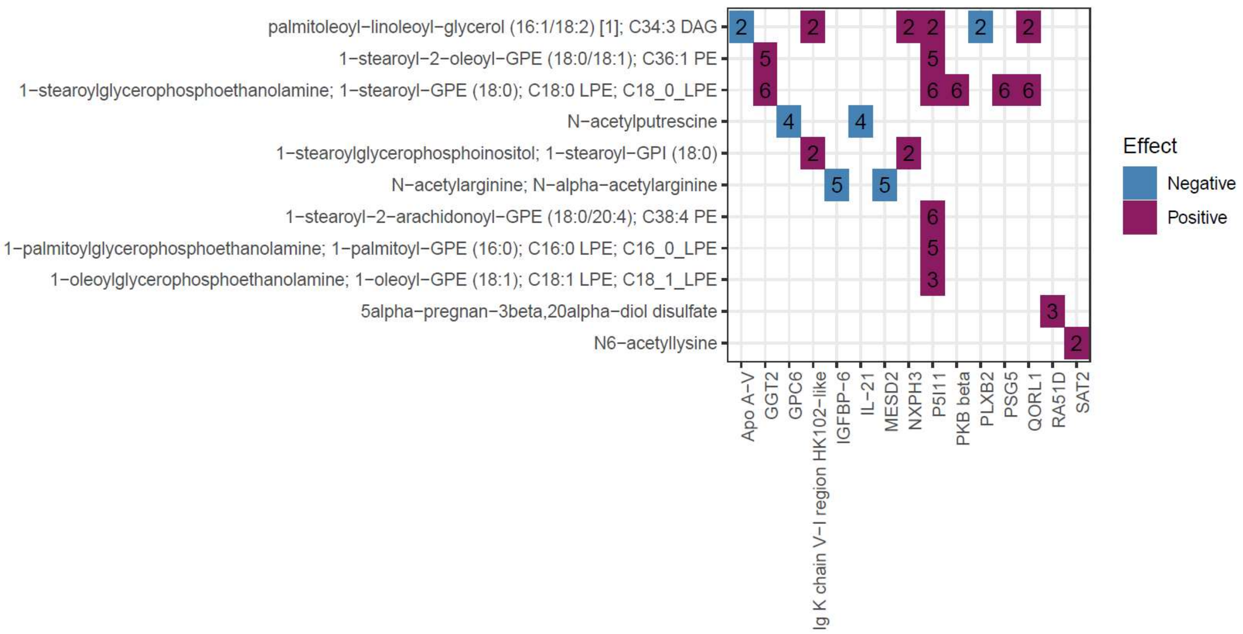

Supplementary Fig. 4. MR results: metabolites effect on pQTL.

Metabolites are provided on the y-axis, pQTLs are provided on the x-axis. The color indicates whether the metabolite increases protein expression (purple), or decreases protein expression (blue). The number on the center of each square indicates the number of variants used to obtain each result.
