# Supplementary material for: Whole-Genome Sequencing Analysis of Human Metabolome in Multi-Ethnic Populations
Source: Nat Commun. 2023 May 30;14:3111. doi: 10.1038/s41467-023-38800-2 (PMC10229598; doi:10.1038/s41467-023-38800-2)
Supplement: Supplementary file 2 — Description of Additional Supplementary Files [file 41467_2023_38800_MOESM2_ESM.pdf]

Description of Additional Supplementary Files

File name: Supplementary Data 1
Description: Trans-Omics for Precision Medicine (TOPMed) Authorship list (April 11, 2023).

File name: Supplementary Data 2
Description: Study information, metabolite measurement methods and genotyping information (a) for studies in the discovery stage; (b) for studies in the replication stage meta-analyses.

File name: Supplementary Data 3
Description: Basic characteristics of the study with regards to age, sex and eGFR (a), metabolites (b), and genetic locis definitions (c).

File name: Supplementary Data 4
Description: Known conditionally independent variant-metabolite associations.
Results are presented for variants reaching two-sided P-value ≤ 3x10-11 (with significance threshold adjusted for multiple comparisons) in the discovery single variant analyses.
Locus ID - metabolite associated genetic regions for each set of the correlated metabolites, containing all statistically significant variants within 500kb from each other, with addition of 500kb to each side of the region (all overlapping regions were merged); Super Pathway - super-pathway to which each respective metabolite belongs; HMDB - HMDB identifier for the metabolite (when available); Metabolite - metabolite for which known association in the region was previously reported; rsID - conditionally independent variants within the region; Gene - either the gene that contains the variant or the closest gene; Consequence - most deleterious functional consequence to the transcript, according to Variant Effect Predictor); EA - effect allele; OA - other allele; EAF - effect allele frequency; Beta - effect size; SE - standard error; PVE - proportion of variance in metabolite explained by a given SNP; Reference - source of the previous report(s) for the respective region (PubMedID, doi, or metabolite is within the Super-pathway, where other metabolites were reported previously for the respective region).

File name: Supplementary Data 5
Description: Novel conditionally independent variant-metabolite associations a) available in replication cohorts, b) not available in replication cohorts.
Results are presented for variants reaching two-sided P-value ≤ 3x10-11 (with significance threshold adjusted for multiple comparisons) in the discovery single variant analyses.
Locus ID - metabolite associated genetic regions for each set of the correlated metabolites, containing all statistically significant variants within 500kb from each other, with addition of 500kb to each side of the region (all overlapping regions were merged); Super Pathway - super-pathway to which each respective metabolite belongs; HMDB - HMDB identifier for the metabolite (when available); Metabolite - metabolite for which known association in the region was previously reported; rsID - conditionally independent variants within the region; Gene - either the gene that contains the variant or the closest gene; Consequence - most deleterious functional consequence to the transcript, according to Variant Effect Predictor); EA - effect allele; OA - other allele; EAF - effect allele frequency; Beta - effect size; SE - standard error; N - number of participants included; PVE - proportion of variance in metabolite explained by a given SNP.

File name: Supplementary Data 6
Description: Replicated novel independent statistically significant single variant-metabolite associations.
Results are presented for variants reaching two-sided P-value <1.02x10-4 (with significance threshold adjusted for multiple comparisons) in the replication single variant meta-analyses.
Note: detailed information for all variants in this table can be found in Supplementary Data 4.

File name: Supplementary Data 7
Description: Generalization of novel findings in pediatric populations.
Results are presented for variants reaching two-sided P-value ≤ 3x10-11 (with significance threshold adjusted for multiple comparisons) in the discovery single variant analyses, which were available in pediatric populations.

File name: Supplementary Data 8
Description: Coding gene-centric analysis results.
Results are presented for gene-metabolite associations reaching two-sided P-value ≤ 1.05x10-9 (with significance threshold adjusted for multiple comparisons).

File name: Supplementary Data 9
Description: Non-coding gene-centric analysis results.
Results are presented for gene-metabolite associations reaching two-sided P-value ≤ 1.05x10-9 (with significance threshold adjusted for multiple comparisons).

File name: Supplementary Data 10
Description: Colocalization analysis for (a) metabolite-associated loci with eQTLs from GTEx V8 and (b) sensitivity analyses.
Results are presented for variants with posterior probability (PPr) > 0.6 for colocalization between metabolite(s) with gene eQTLs in tissue(s).
rsID - causal variant shared across traits; Gene - either the gene that contains the candidate causal variant or the closest gene; Posterior probability - probability that signals colocalize; Posterior probability of regional colocalization - probability that signals colocalize to the region, but may not colocalize to single candidate causal variant; Posterior probability explained by SNP - proportion of posterior probability (column G) explained by the candidate causal variant; Expressed gene (eQTL) - Gene with which eQTL association was observed; Tissues - tissues in which eQTL colocalises with the metabolite; Metabolite(s) - metabolite(s) that colocalize with the eQTL, and which are associated with the novel independent globalized variant, belonging to the tested genetic locus; Genetic locus start - the first position of the tested genetic locus; Genetic locus end - the last position of the tested genetic locus.

File name: Supplementary Data 11
Description: Pathway Analysis Results.
Results are presented for pathways with two-sided P-value ≤ 2.70x10-8, accounting for 28,438 Biological Process terms in Gene Ontology annotations and 65 metabolites

File name: Supplementary Data 12
Description: Mendelian Randomization results for 1,801 FinnGen traits, reaching statistical significance (Inverse Variance Weighted meta-analysis two-sided P-value<1.51e-07, adjusting for multiple comparisons) for loci with more than 1 IV available.

File name: Supplementary Data 13
Description: Mendelian Randomization Results for 3,283 SOMAMER pQTLs predicting metabolite levels reaching statistical significance (Inverse Variance Weighted meta-analysis two-sided P-value<4.93e-07, adjusting for multiple comparisons) for loci with more than 1 IV available

File name: Supplementary Data 14
Description: Mendelian Randomization Results for 3,283 SOMAMER pQTLs reaching statistical significance (Inverse Variance Weighted meta-analysis two-sided P-value<1.51e-07, adjusting for multiple comparisons) for loci with more than 1 IV available.
