# Supplementary material for: Whole-Genome Sequencing Analysis of Human Metabolome in Multi-Ethnic Populations
Source: Nat Commun. 2023 May 30;14:3111. doi: 10.1038/s41467-023-38800-2 (PMC10229598; doi:10.1038/s41467-023-38800-2)
Supplement: Supplementary file 5 — Reporting Summary [file 41467_2023_38800_MOESM5_ESM.pdf]

nature portfolio

Corresponding author(s): Bing Yu

Last updated by author(s): Apr 13, 2022

Reporting Summary

Nature Portfolio wishes to improve the reproducibility of the work that we publish. This form provides structure for consistency and transparency in reporting. For further information on Nature Portfolio policies, see our Editorial Policies and the Editorial Policy Checklist.

Statistics

For all statistical analyses, confirm that the following items are present in the figure legend, table legend, main text, or Methods section.

n/a	Confirmed	
☐	☒	The exact sample size (n) for each experimental group/condition, given as a discrete number and unit of measurement
☐	☒	A statement on whether measurements were taken from distinct samples or whether the same sample was measured repeatedly
☐	☒	The statistical test(s) used AND whether they are one- or two-sided<br>*Only common tests should be described solely by name; describe more complex techniques in the Methods section.*
☐	☒	A description of all covariates tested
☐	☒	A description of any assumptions or corrections, such as tests of normality and adjustment for multiple comparisons
☐	☒	A full description of the statistical parameters including central tendency (e.g. means) or other basic estimates (e.g. regression coefficient) AND variation (e.g. standard deviation) or associated estimates of uncertainty (e.g. confidence intervals)
☐	☒	For null hypothesis testing, the test statistic (e.g. F, t, r) with confidence intervals, effect sizes, degrees of freedom and P value noted<br>*Give P values as exact values whenever suitable.*
☒	☐	For Bayesian analysis, information on the choice of priors and Markov chain Monte Carlo settings
☒	☐	For hierarchical and complex designs, identification of the appropriate level for tests and full reporting of outcomes
☐	☒	Estimates of effect sizes (e.g. Cohen's d, Pearson's r), indicating how they were calculated

Our web collection on statistics for biologists contains articles on many of the points above.

Software and code

Policy information about availability of computer code

Data collection	Discovery was performed in up to 11,840 participants from five cohorts - the Atherosclerosis Risk in Communities study (ARIC), Hispanic Community Health Study / Study of Latinos (HCHS/SOL), Framingham Heart Study (FHS), Cardiovascular Health Study (CHS), and Multi-Ethnic Study of Atherosclerosis (MESA).<br>Replication was performed in up to five cohort studies (independent participants from FHS, Women's Health Initiative [WHI], Jackson Heart Study [JHS], FENLAND, TwinsUK), including 2,466 AA and 15,619 individuals of European ancestry, for a total sample size of up to 18,085 individuals.<br>The information on participating cohorts, as well as metabolite measurement methods and genotyping information is presented in Supplementary Table 1. Demographics of study participants, the biochemical name, pathway and missingness for each metabolite are summarized in Supplementary Table 2.
Data analysis	Single variant tests and conditional analyses - GENESIS version 2.12.2<br>Inverse-variance weighted meta-analysis of single variant summary statistics - meta version 4.18-0 R package.<br>Gene-centric rare variant analyses were performed using STAAR package v.0.9.5<br>Co-localisation analysis was performed using HyPrColoc package in R version 1.0<br>The gene-level association analysis - MAGMA tool version 1.09a<br>Gene networks associated with each metabolite - dmGWAS version 2.7<br>The ORA was performed by using the WebGestalt R package version 2019.<br>Mendelian Randomization analyses - TwoSampleMR 0.5.6 R package, MR-PRESSO version 1.0

For manuscripts utilizing custom algorithms or software that are central to the research but not yet described in published literature, software must be made available to editors and reviewers. We strongly encourage code deposition in a community repository (e.g. GitHub). See the Nature Portfolio guidelines for submitting code & software for further information.

Data

Policy information about availability of data

All manuscripts must include a data availability statement. This statement should provide the following information, where applicable:

- Accession codes, unique identifiers, or web links for publicly available datasets
- A description of any restrictions on data availability
- For clinical datasets or third party data, please ensure that the statement adheres to our policy

Individual whole-genome sequence data from the TOPMed program are available through dbGaP. The dbGaP accession numbers are: Atherosclerosis Risk in Communities (ARIC) phs001211, Cardiovascular Health Study (CHS) phs001368, Framingham Heart Study (FHS) phs000974, Multi-Ethnic Study of Atherosclerosis (MESA) phs001416, and Hispanic Community Health Study - Study of Latinos (HCHS-SOL) phs001395. Data in dbGaP can be downloaded by controlled access with an approved application submitted through their website: https://www.ncbi.nlm.nih.gov/gap. Individual metabolite data are available via request per each study policy.

Field-specific reporting

Please select the one below that is the best fit for your research. If you are not sure, read the appropriate sections before making your selection.

☒ Life sciences ☐ Behavioural & social sciences ☐ Ecological, evolutionary & environmental sciences

For a reference copy of the document with all sections, see nature.com/documents/nr-reporting-summary-flat.pdf

Life sciences study design

All studies must disclose on these points even when the disclosure is negative.

Sample size	To explore the genetic architecture of the human metabolites, we collected data from five cohorts, as described above (up to 11,840 participants), which had both the Whole Genome Sequencing data, and the circulating Metabolites data.
Data exclusions	Quality Control for the Whole Genome Sequencing was performed prior to the current study (detailed quality control procedures are described at https://www.nhlbiwgs.org/topmed-whole-genome-sequencing-methods-freeze-8).<br>For single variant analysis, variants with minor allele frequency < 0.5%, sample size < 200 or minor allele count (MAC) < 5 were excluded.<br>For gene-based analysis, gene-metabolite associations with the sample size of less than 5,000 and cumulative MAC of less than 100 were excluded.
Replication	For replication, summary association statistics were requested (for the novel variant-metabolite associations with P < 3x10-11) from three cohorts: FHS (2,969 EAs), WHI (1,328 EA), and JHS (2,466 AAs). Additionally, we obtained publicly available summary statistics from 9,363 European FENLAND participants 21 and 1,959 EUR TwinsUK participants 1. In total, up to 18,085 individuals (including 15,619 European ancestry participants and 2,466 AA participants) were available for replication meta-analysis. Study-specific characteristics are provided in Supplementary Table 1B.<br>We also requested summary statistics for 1,985 novel variant-metabolite associations from two children studies - Childhood Asthma Management Program (CAMP) and Genetic Epidemiology of Asthma in Costa Rica (CRA) (Supplementary Table 5).
Randomization	N/A
Blinding	N/A

Reporting for specific materials, systems and methods

We require information from authors about some types of materials, experimental systems and methods used in many studies. Here, indicate whether each material, system or method listed is relevant to your study. If you are not sure if a list item applies to your research, read the appropriate section before selecting a response.

Materials & experimental systems

n/a	Involved in the study	
☒	☐	Antibodies
☒	☐	Eukaryotic cell lines
☒	☐	Palaeontology and archaeology
☒	☐	Animals and other organisms
☐	☒	Human research participants
☒	☐	Clinical data
☒	☐	Dual use research of concern

Methods

n/a	Involved in the study	
☒	☐	ChIP-seq
☒	☐	Flow cytometry
☒	☐	MRI-based neuroimaging

Human research participants

Policy information about studies involving human research participants

Population characteristics	In the discovery analyses we analysed up to 15,660,619 common (Minor Allele Frequency [MAF] ≥ 5%), low-frequency (1% < MAF < 5%) and rare (MAF ≤ 1%) variants belonging to autosomal chromosomes and the X chromosome for association with 1,666 rank-normalized circulating metabolites in up to 11,840 participants (mean age at 56.7 years old, 57% women) from a pooled sample of 1,843 African American (AA), 5,938 European American (EA) and 4,059 Hispanic (HIS) participants from the Atherosclerosis Risk in Communities study (ARIC), Hispanic Community Health Study / Study of Latinos (HCHS/SOL), Framingham Heart Study (FHS), Cardiovascular Health Study (CHS), and Multi-Ethnic Study of Atherosclerosis (MESA) (Methods). For replication analysis, we obtained summary statistics from up to five cohort studies (independent participants from FHS, Women's Health Initiative [WHI], Jackson Heart Study [JHS], FENLAND, TwinsUK), including 2,466 AA and 15,619 individuals of European ancestry, for a total sample size of up to 18,085 individuals (Methods). The information on participating cohorts, as well as metabolite measurement methods and genotyping information is presented in Supplementary Table 1. Demographics of study participants, the biochemical name, pathway and missingness for each metabolite are summarized in Supplementary Table 2. The study design, applied statistical and functional analyses, and an overview of the known and novel findings are displayed in Fig. 1.<br><br>Blood samples were sequenced on the Illumina HiSeq X; for MESA and FHS, sequencing was performed by the Broad Institute of MIT and Harvard; for CHS and HCHS/SOL - by the Baylor College of Medicine Human Genome Sequencing Center, while for ARIC - by both centers. Variants calling was completed using the GotCloud pipeline 68.<br><br>Details of the metabolites measurements are provided in Supplementary Table 1 and Supplementary Information, while for the previously published studies, these can be found in the respective manuscripts 1; 21. In brief, blood samples were collected in participating studies, processed and stored at -70 °C since collection. Overall, 1,666 metabolites were measured by untargeted, gas and/or liquid chromatography-mass spectrometry-based quantification protocol (Supplementary Table 2) 66; 67. In HCHS/SOL and ARIC, metabolites were measured by Metabolon Inc. (Durham, NC) platform. For CHS, FHS, JHS MESA, and WHI, metabolites were measured by Broad Institute.
Recruitment	Recruitment procedures for each of the participating cohorts were described previously elsewhere.
Ethics oversight	All the participating studies were approved by corresponding institutional review boards, and all participants provided written informed consent.

Note that full information on the approval of the study protocol must also be provided in the manuscript.
